# Increasing integrated testing in community settings through interventions for change, including the Spring European Testing Week

**DOI:** 10.1186/s12879-021-06555-0

**Published:** 2021-09-13

**Authors:** Nadia Gasbarrini, Davor Dubravić, Lauren Combs, Arian Dišković, Magdalena Ankiersztejn-Bartczak, Francesca Colaiaco, Iwona Wawer, Piotr Wysocki, Magdalena Rosińska, Anna Marzec-Boguslawska, Ben Collins, Daniel Simões, Marie Louise Jakobsen, Dorthe Raben

**Affiliations:** 1 Fondazione Villa Maraini, Rome, Italy; 2 Croatian Association for HIV and Viral Hepatitis (HUHIV), Zagreb, Croatia; 3 CHIP, Centre of Excellence for Health, Immunity and Infections,, Rigshospitalet, University of Copenhagen, Blegdamsvej 9, 2100 Copenhagen Ø, Denmark; 4 Foundation for Social Education, Warsaw, Poland; 5 Associazione Della Croce Rossa Italiana (CRI), Rome, Italy; 6 National AIDS Centre, Agency of the Ministry of Health, Warsaw, Poland; 7 Department of Epidemiology of Infectious Diseases and Surveillance, National Institute of Public Health - National Institute of Hygiene, Warsaw, Poland; 8 ReShape/International HIV Partnerships, London, UK; 9 EPIUnit - Instituto de Saúde Pública, Universidade do Porto, Porto, Portugal

**Keywords:** Testing, Integrated testing, HIV, Hepatitis, Sexually transmitted infections, Community

## Abstract

**Background:**

Maximising access to testing by targeting more than one infection is effective in identifying new infections in settings or populations. Within the EU funded Joint Action INTEGRATE, this paper examined the feasibility and impact of expanding integrated testing for HIV, hepatitis C (HCV), chlamydia, gonorrhoea and/or syphilis in four community-based pilots through targeted interventions in Croatia, Italy and Poland and the Spring European Testing Week since community settings are key in detecting new infections and reaching key populations.

**Methods:**

Pilots led by local INTEGRATE partners prioritised testing for other infections or key populations. The Croatian pilot expanded testing for men who have sex with men to syphilis, chlamydia and gonorrhoea. Italian partners implemented a HIV and HCV testing/information event at a migrant centre. A second Italian pilot tested migrants for HIV and HCV through outreach and a low-threshold service for people who use drugs. Polish partners tested for HIV, HCV and syphilis among people who inject drugs in unstable housing via a mobile van. Pilots monitored the number of individuals tested for each infection and reactive results.

The pilot Spring European Testing Week from 18 to 25 May 2018 was an INTEGRATE-driven initiative to create more testing awareness and opportunities throughout Europe.

**Results:**

The Croatian pilot found a high prevalence for each syphilis, chlamydia and gonorrhoea respectively, 2.1%, 12.4% and 6.7%. The Italian migrant centre pilot found low proportions who were previously tested for HIV (24%) or HCV (11%) and the second Italian pilot found an HCV prevalence of 6.2%, with low proportions previously tested for HIV (33%) or HCV (31%). The Polish pilot found rates of being previously tested for HIV, HCV and syphilis at 39%, 37%, and 38%, respectively. Results from the Spring European Testing Week pilot showed it was acceptable with increased integrated testing, from 50% in 2018 to 71% in 2019 in participants.

**Conclusions:**

Results show that integrated testing is feasible and effective in community settings, in reaching key populations and minimising missed testing opportunities, and the pilots made feasible because of the European collaboration and funding. For sustainability and expansion of integrated community testing across Europe, local government investment in legislation, financial and structural support are crucial.

## Background

Integrated testing (testing for more than one infection) for HIV, hepatitis B (HBV) and C (HCV) virus and sexually transmitted infections (STIs) has been identified and supported by global and European organisations, including the European Commission, the European Centre for Disease Prevention and Control (ECDC) and the World Health Organization (WHO) [1–4] as an effective method to increase testing coverage and identify new infections, especially among key population groups due to overlapping modes of transmission and high prevalence of co-infection. Integrating testing can reduce missed opportunities for earlier diagnosis, maximise the use of existing infrastructure and resources while minimising delays in linkage to care for multiple infections, utilising a simplified, people-centred rather than disease-centred approach [1].

INTEGRATE [5] is an European Commission co-funded Joint Action (2017–2020) which aims to examine the feasibility and impact of expanding integrated testing, including in community settings. INTEGRATE partners or affiliated community-based organisations from Croatia, Italy and Poland implemented four pilot projects aiming to provide integrated testing activities targeting HIV, HBV, HCV and/or STIs to optimise testing coverage in key populations including men who have sex with men (MSM), migrants and people who inject and/or use drugs (PWID/PWUD). All partners provide services for key populations and most provide integrated testing (for at least two or more infections) carried out by health care staff. However, the national and local responses to HIV, HBV, HCV and STIs in the three countries vary widely due to differing epidemiological, regulatory and financial landscapes.

In Croatia, there is a national response strategy for HIV, however, strategic guidance is lacking for both the detection of HBV, HCV and testing by trained lay providers. Current legislation is also punitive against certain vulnerable groups, and key populations report widespread discrimination and stigma in the formal health care system [6, 7].

In Italy, although national testing policies for HIV, HBV and HCV are comprehensive and promote widespread accessibility [8, 9], in practice, policy recommendations have not been implemented [10], and key guidance on lay provider testing is missing. Hospital settings are the most frequently used and best-known setting where people can obtain testing for HIV, HBV, HCV and STIs [11], however, stigma, discrimination and unfair treatment by health care providers has been reported by key populations [12, 13]. There have been growing efforts to expand access to testing outside of formal health care settings and recent national data found it to be widely acceptable especially among key groups [14].

In Poland, the national HIV response strategy has a strong partnership between the government and community network [15], however, due to the siloed-health care system, where each infection is separately addressed, Poland lacks national screening programmes for viral hepatitis and STIs with no regulation for integrated testing for viral hepatitis and STIs in community settings. While nonmedical sites are the preferred service setting key population groups because of the anonymity and non-judgemental approach, lay providers are not allowed to test.

Additionally, as another method of expanding integrated testing opportunities, INTEGRATE, in collaboration with the European Testing Week (ETW) initiative and the European Liver Patients’ Association, piloted a Spring-version of European Testing Week (SETW) which took place 18–25 May 2018. Since 2015, ETW occurs annually during the last week of November as a HIV/HBV/HCV testing awareness campaign, encouraging partners in civil society, health care, and policy institutions in the WHO European Region to unite to promote awareness of and increase coverage of HIV, HBV, and HCV testing. The SETW was piloted to assess the feasibility of implementing ETW twice a year during a different season, its impact on increasing integrated activities for HIV/HBV/HCV and reaching key populations. This article will present results from the pilot SETW and four pilot activities to describe the impact of expanding testing accessibility.

## Methods

### Pilot development

All partners routinely perform testing and assessed their client’s needs to determine the most relevant activities to implement in the pilots including infections to test, groups to target, duration of the activity, selection criteria and recruitment strategies. Universally, individuals aged ≥ 18 years were included in the pilots and consent to test was obtained according to local EC/IRB regulation.

The INTEGRATE partner, Croatian association for HIV and viral hepatitis (HUHIV), routinely tests for HIV and HCV for MSM through their Checkpoint in Zagreb. For the pilot, the test offer for MSM was expanded to include syphilis, chlamydia and gonorrhoea. All clients seen were offered additional testing upon risk assessment. HUHIV obtained additional funding from the local government and received donated chlamydia/gonorrhoea tests. It was implemented in collaboration with the Clinic of Infectious Diseases ‘Fran Mihaljevic’ who processed the results and provided staff training.

Italian INTEGRATE partners, Associazione della Croce Rossa Italiana (CRI) and Fondazione Villa Maraini (FVM), collaborated to conduct a 1-day testing/informative session at a migrant centre managed by CRI in Rome about HIV/HCV (general information, ways of transmission and risk behaviours, diagnosis and treatment) and substance abuse. Two preparatory meetings were held with centre staff to define the intervention. Participants were recruited to the informative session by centre staff and informative leaflets translated in English, Arabic, French, Turkish and Urdu prior to the event. The session was translated by cultural mediators in French, Arabic and Turkish. After the session, voluntary free testing was offered to all clients at the centre. After providing informed consent, clients responded to a risk-behaviour questionnaire interview, available only in Italian and English. Attendance to the informative session was not mandatory to access testing.

FVM also routinely offers combined testing for HIV and HCV for PWUD, MSM, sex workers and other key groups, both in its medical centre for PWUDs (drop-in, night shelter and opioid substitution treatment centre) and in an outreach street unit. For the second pilot, FVM conducted targeted testing for migrants in these two settings. To initiate testing, FVM staff informed potential clients on the importance of getting tested and provided informed consent forms and the risk-behaviour questionnaires in English, Romanian or Arabic.

INTEGRATE partner, National AIDS Centre (NAC) in Poland in cooperation with the Foundation for Social Education (FES), a community-based non-governmental organisation (NGO), piloted a mobile service which provided integrated testing for HIV, HCV and syphilis targeting PWID experiencing homelessness in addition to harm reduction, medical care and social support services. The mobile van travelled to four vacant properties in Warsaw and recruited participants via convenience sampling, respondent driven sampling (RDS) and/or street recruitment. All who were interested in any of the other services were offered testing.

### Pilot monitoring

Across all pilots, monitoring was measured by a basic set of indicators including at minimum: the infection tested, the total number of individuals tested for each infection during the pilot and number of reactive results. These indicators were selected as they were universally collected across all pilots and all partners were able to collect this data. Optional indicators included the number of individuals with a known positive status who were tested and the number of individuals who were previously tested within the past 12 months. All pilots collected anonymised data which was submitted in an aggregated format via an online form on REDCap, Excel or a report.

All pilots are established testing sites and no specific ethical approval was required for the additional pilot testing activities. Aggregated data on testing collected for this article contained no personal identifiers and the client surveys were conducted anonymously.

### Spring European Testing Week

To participate in the SETW, interested organisations sign-up on the ETW website with the intention to take action to organise their own local, national or regional activities to increase HIV/HBV/HCV testing awareness and access. There is no minimum requirement to participate and organisations volunteer their time to organise activities in their community.

At the completion of ETW, all participants are invited to complete an online evaluation which measures the types of activities, targeted key groups, details on activities, satisfaction with ETW and challenges. Organisations who conducted testing are invited to submit aggregated data on people tested, reactive results and linkage to care via an Excel form. For this article, a comparison was performed between post-ETW data from November 2017 with SETW data from 2018 and 2019 to evaluate the impact of conducting ETW during Spring.

## Results

### Expansion of testing

For pilot partners who expanded existing services to include integrated testing for other infections (Table 1), there was a notable number of previously undiagnosed infections detected (Table 2). Croatian partner, HUHIV, tested clients for syphilis (N = 144), chlamydia (N = 89) and gonorrhoea (N = 89), and found high prevalence for each infection respectively, 2.1%, 12.4% and 6.7%. The percentage reporting previous testing for chlamydia (15%) and gonorrhoea (15%) was low. Routine testing for HIV and HCV also yielded positive results with prevalence rates at 0.6% and 1.0%, respectively, with low percentages reported being tested within the past 12 months, 21% and 18%. All clients who received a HIV, HCV and/or syphilis reactive result were offered additional post-test counselling with linkage to confirmatory testing and/or treatment at a 100% rate, while linkage following a positive result for chlamydia and/or gonorrhoea was not systematically tracked. Chlamydia and gonorrhoea results were processed in a laboratory and clients could choose to receive results via in-person or e-mail.

**Table 1:** Description of partner sites and pilot activities

COUNTRY	Organisation	Type of setting	Baseline			Pilot activities			
			Main populations served	Testing available for:	Personnel allowed to provide testing	Description of activity/intervention	Targeted population	Tested infection	Type of test
Croatia	Croatian association for HIV and viral hepatitis (HUHIV)	Community NGO	PLHIV, people with HCV, youth, women, MSM, PWID, sex workers	HIV, HCV, syphilis	Doctors	~ 3-month pilot at a community-based NGO to implement integrated testing for clients	MSM	HIV	RDT
								HCV	RDT
								Syphilis	RDT
								Chlamydia	Molecular test
								Gonorrhoea	Molecular test
Italy	Croce Rossa Italiana (CRI)	Community NGO	Youth	HIV, HCV	Doctors	(In collaboration with FVM) 1-day testing and information event at a migrant centre	Migrants	HIV	RDT
								HCV	RDT
	Fondazione Villa Maraini Onlus (FVM)	Drug rehabilitation centre, outreach	PLHIV, people with HCV, PWUD	HIV, HCV, syphilis	Doctors, social workers	Targeted testing for migrants through outreach street unit and low-threshold services over 12 months	Sex workers, migrants, PWUD	HIV	RDT
								HCV	RDT
Poland	Fundacja Edukacji Społecznej/ Foundation for Social Education (FES)^a^	VCT outreach, NGO	PLHIV, women, youth, MSM, PWID	HIV, HCV, syphilis	Doctors	6-days of street outreach through a mobile unit at 4 vacant properties providing integrated testing	PWID experiencing homelessness	HIV	RDT
								HCV	RDT
								Syphilis	RDT

^a^On behalf of the National AIDS Centre (NAC)

**Table 2:** Results from pilot integrated testing activities

Organisation	Tested infection	No. of individuals tested	No. of individuals with reactive screening (No. of known positive status)	Newly diagnosed infections per 100 tests	No. previously tested within the past 12 months
HUHIV	HIV	341	2 (−)	0.6%	72 (21%)
	HCV	197	2 (−)	1.0%	36 (18%)
	Syphilis	144	3 (−)	2.1%	-
	Chlamydia	89	11 (−)	12.4%	13 (15%)
	Gonorrhoea	89	6 (−)	6.7%	13 (15%)
FVM/CRI	HIV	64	0 (−)	0.0%	15 (24%)
	HCV	64	0 (−)	0.0%	7 (11%)
FVM	HIV	250	0 (−)	0.0%	82 (33%)
	HCV	242	15 (−)	6.2%	75 (31%)
FES	HIV	95	0 (6)	0.0%	39 (39%)
	HCV	76	5 (20)	6.6%	36 (37%)
	HBV	–	–	–	4 (4%)
	Syphilis	100	0 (1)	0.0%	38 (38%)

At the CRI/FVM-hosted testing/informative event at the migrant centre in Rome, 26 clients attended the session, while 64 clients were tested for both HIV and HCV with no reactive results. Data from the risk assessment questionnaire, showed low rates of clients that previously tested for HIV (24%) and for HCV (11%).

Through FVM’s pilot in their low threshold centre and street outreach, 250 individuals were tested for HIV and 242 for HCV. No new cases of HIV were detected, while an HCV prevalence of 6.2% was found. Regarding having previously tested within the past year, the reported rates were low for both HIV (33%) and HCV (31%). Those who received a reactive result were linked to the Infectious Diseases Clinic of Policlinico Tor Vergata in Rome.

In Poland, 101 clients were tested via the mobile van, which found an HCV prevalence of 6.6% for new infections and no new detection of HIV or syphilis. Regarding previous testing in the past year, the found rates were low for HIV (39%), HCV (37%) and syphilis (38%). Clients who received a reactive result were directed to specialised clinics. Since the clients were anonymous, there was no possibility for follow-up of linkage to care.

### Spring European Testing Week

The number of organisations participating in the pilot SETW in 2018 and in 2019 was 104 and 136, with response rates to the survey of 32% and 36%, respectively (Table 3). Results from the pilots showed increases in activities targeted for more than one infection, including HIV, HBV, HCV, and in 2019, also for STIs and tuberculosis (TB), with an increase from 50 to 71% of participating organisations reporting combined activities. More than 60% of respondents reported an estimated increase in testing of at least 50% or more during ETW compared to a normal week.

**Table 3:** Results from Spring European Testing Week 2018 & 2019 compared to the 2017 November ETW

Year	No. of registered organisations	Survey response rate	Percentage reporting single infection targeted ETW activities	Percentage reporting combined (more than one infection) ETW activities	Overall estimated reported increase in testing of at least 50% during ETW^b^	No estimated increase in testing during ETW^b^
2017 (November ETW)	640	24% (155)	50%	50%	82%	18%
2018 SETW (pilot)	104	32% (33)	36%	64%	66%	34%
2019 SETW	136	36% (49)	29%	71%^a^	68%	32%

^a^For 2017 and the 2018 pilot, respondents reported on combined activities for HIV/HBV/HCV. In 2019, respondents were able to report combined activities for the three infections in addition to syphilis, gonorrhoea, chlamydia and tuberculosis

^b^Respondents are asked to report approximate increase (rough estimate) in tests performed at organisation/clinic/hospital during ETW compared to an average week each for HBV, HCV and HIV by the following scale: > 200% increase, 100–200% increase, 50–100% increase, Up to 50% increase, No increase. The included percentage is the pooled average of all reporting at least a 50% increase in testing

## Discussion

These pilot activities show that testing for more than one infection is feasible in community settings, has the potential to increase case finding for multiple infections and reduce missed opportunities for testing in key populations who do not typically access traditional health services. All pilots reached key groups where two-thirds were not recently tested for the infections included in the pilots. While recommendations for frequent/repeat testing vary nationally across Europe taking into consideration risk-group, service setting and specific infection [3, 4], studies among key populations have shown that repeat testing rates are still low. For STIs, studies in the UK and the Netherlands [16, 17] show an estimated proportion of MSM who have had a history of HIV and/or STI testing within the past 12 months ranged from 33 to 36%, which is higher than what was found in the pilots. A Croatian study which assessed factors for repeat HIV testing in a community setting found that 70% of MSM in their study and 57% of people with a history of injecting drug use were most likely to have had a history of an HIV test [18]. These results support recommendations from the WHO and ECDC [3, 4] to implement and support integrated and repeat testing. However, the pilots’ success was achieved through temporary solutions within the context of a regional-European project and sustainability of these interventions requires local and national support from relevant stakeholders, including policy makers, laboratories and clinical care structures.

When implementing the pilot activities, expansion of integrated testing was conditional based on a European project, but no plans for incorporation into permanent practice was discussed with stakeholders. HIV testing is more widely available allowing for more flexibility in community-led service implementation, while HBV, HCV and/or STIs do not have the same legal and regulatory provisions and are embedded in traditional health care settings. WHO recommends integrating the HIV, viral hepatitis and STI responses in order to accelerate the elimination of these infections by 2030 [19, 20], however, in both Croatia and Poland, each infection has its own set of regulations where solutions that can be implemented for HIV, cannot be implemented for the other infections under current legal framework.

All partners were impacted by the lack of regulation and national policy to allow trained lay providers to conduct testing in community settings. Although trained lay provider testing is proven to be effective in increasing HIV testing [21, 22] and is recommended by international guidelines [3, 4], countries have been slow to adapt policies to support its rollout. Additionally, the requirement to employ medical professionals to conduct testing constitutes a financial burden for those unable to apply cost-effective task shifting with lay providers [22], which is proven to increase availability and scale up of testing at controlled costs. Lay provider or peer-driven testing interventions have also been proven to be effective in engaging, recruiting and supporting clients, especially key groups disproportionately affected by these infections [23].

Despite the positive results, the partners faced implementation issues that hindered opportunities to maximise access to the pilot services. In Croatia, HUHIV was legally permitted to provide testing for chlamydia and gonorrhoea but only for MSM clients, which excluded all other clients in need. Additionally, results for chlamydia and gonorrhoea tests took two workdays, while results for HIV, HCV and syphilis were available on-site same day.

In Italy, the pilot targeting migrant populations faced cultural challenges due to lack of knowledge and interest in HIV and HCV [24], related to cultural norms of many migrants’ countries of origin, where there is hesitancy to discuss these infections and risk-behaviours [25, 26]. Additionally, although national policy stipulates migrants with or without legal status have access to primary health care (which includes testing and harm reduction services), in practice it has been reported that health professionals obstruct migrants’ access citing bureaucratic reasons and migrants without documented legal status experience discrimination [13]. Italian INTEGRATE partner, FVM, is among the only centres in Rome that provides OST, other therapies and free testing for all clients, without requiring proof of legal residency. In order to support duplication of the pilot in other organisations, more political and provider-will are needed.

The benefits of community testing has been widely proven and recommended [3, 4, 21, 22], however linkage to confirmatory testing and care following a reactive result, especially in the case of anonymous testing, proved to be a challenge for the pilots for the newly introduced tests. For HUHIV, since chlamydia and gonorrhoea are primarily managed through the formal health care system in Croatia and tests were anonymous, not all clients were systematically tracked followed testing. In Italy, although the pilots successfully provided testing to migrants, organisational and administrative issues act as barriers for migrants to access health care [27] and studies have shown that high proportions of undocumented migrants are lost to follow-up [28]. Lastly, in Poland, linkage from anonymous HIV testing in outreach settings makes follow-up on linkage to care not possible, while for hepatitis follow-up and treatment, clients must go to their primary care provider for referral, which is a barrier since many key groups are not comfortable with engaging with the formal health care system [15].

For the Spring European Testing Week, the pilot in 2018 examined the impact of implementing ETW biannually and during a different time of the year and found that it was feasible and acceptable. Widespread awareness campaigns have been proven to be effective in promoting sexual health and preventing HIV and other STIs [29, 30] and ETW has established itself as a well-known European campaign since 2013. Through building upon its existing platform, the pilot was successful in increasing integrated activities for HIV/HBV/HCV and testing coverage during a different time period creating more opportunities for testing for multiple infections. By focussing on integrated activities, there is the potential of closing the gaps on missed opportunities for awareness and testing for other infections.

There are several limitations to this article. Specific to the pilot testing activities, due to the diverse circumstances of the infections in each pilot country, the pilot partners determined their own interventions which made a universal comparison of activities unattainable. Additionally, the interventions were implemented at varying intervals (ranging from a 1-day event to 12 months). Both testing and survey data collection was also limited, and recruitment was primarily conducted through convenience sampling with small overall sample sizes. However, the aim was to assess feasibility and potential benefit of implementing integrated interventions in the real world that are relevant to national testing strategies.

The data from Spring ETW is also limited. Although all ETW participants are asked to complete the online survey, it captures only a small percentage of the activities being organised. Furthermore, not all participants conduct testing activities and of those who do, some are unable to provide data or feedback. The survey is also provided in English which can be a barrier for completion. Lastly, the survey length can act as a barrier since respondents may not have allotted time to complete.

## Conclusions

This article shows that integrated testing outside of traditional health care settings is a feasible and an effective method to increase testing coverage and meet the needs of key populations who experience a disproportionate burden of HIV, HBV, HCV and STIs. The article found low rates of those previously tested within the past year among key populations and found that expanding integrated testing offer in community and outreach settings creates more opportunities to test for multiple infections with the potential of minimising extra costs due to utilising existing structures. National testing strategies should support the scale-up of integrated testing to maximise all opportunities of contact to provide people-centred care, minimise missed opportunities for testing and ensure that more people are aware of their status. The pilots emphasise the need for clear provision in national testing strategies to legislatively and financially support integrated testing in community settings and allowance of trained lay providers to provide testing to maintain sustainability and minimise reliance on time-limited funding. Community representatives should contribute to the development of these strategies to ensure effectiveness and sustainability and local cooperation between all service providers including policy makers, health care providers, laboratories and community-based services is crucial for the success of integrated testing programmes and securing linkage to care.

## Data Availability

All relevant data are within the article and will not be made available.

## Abbreviations

CHIP: Centre of Excellence for Health, Immunity and Infections
CRI: Associazione della Croce Rossa Italiana
ECDC: European Centre for Disease Prevention and Control
ETW: European Testing Week
FES: Fundacja Edukacji Społecznej/Foundation for Social Education
FVM: Fondazione Villa Maraini
HBV: Hepatitis B virus
HCV: Hepatitis C virus
HIV: Human immunodeficiency virus
HUHIV: Croatian association for HIV and viral hepatitis
JA: Joint Action
MSM: Men who have sex with men
NAC: National AIDS Centre
OST: Opioid substitution therapy
PWID: People who inject drugs
PWUD: People who use drugs
RDS: Respondent-driven sampling
RDT: Rapid Diagnostic Test
SETW: Spring European Testing Week
STI: Sexually transmitted infection
TB: Tuberculosis
WHO: World Health Organization
